# Coping strategies in postpartum women: exploring the influence of demographic and maternity factors

**DOI:** 10.1186/s12905-023-02751-z

**Published:** 2023-11-08

**Authors:** Amira Alshowkan, Emad Shdaifat, Fatimah Abdullah Alnass, Friyal Mubarak Alqahtani, Nora Ghalib AlOtaibi, Nagla Saleh AlSaleh

**Affiliations:** 1 Community Nursing Department, College of Nursing, Imam Abdulrahman Bin Faisal University, P. O. Box 1982, Dammam, Saudi Arabia; 2 Fundamental of Nursing Department, College of Nursing, Imam Abdulrahman Bin Faisal University, P. O. Box 1982, Dammam, Saudi Arabia

**Keywords:** Coping Behavior, Postpartum Period, Demography, Maternal health, Problem solving

## Abstract

**Background:**

Postpartum depression is a frequent mental health issue that affects many women due to this stressful phase. The aim of the study is to gain insight into the coping strategies employed by postpartum women and to explore how these strategies are influenced by various demographic and maternity factors.

**Methods:**

The study adopted a quantitative, cross-sectional design. Data were collected from 239 postpartum women receiving care at a Gynecology and Obstetrics Clinic using self-reported tools, which include the Brief COPE survey and the socio-demographics and obstetric/maternal history form from October 2022 to April 2023.

**Results:**

The study findings indicate that individuals aged over 40 tend to use more emotional-focused coping compared to those aged 21–30 (p = 0.002) and 31–40. Additionally, both genders of children were associated with more emotional-focused coping (p = 0.007) compared to only having boys. Cesarean section delivery (p = 0.001) was associated with more avoidant-focused coping than normal vaginal delivery. Avoidant-focused coping was significantly predicted by problem-focused coping (p < 0.001), emotional-focused coping (p = 0.034), age (p = 0.003), and gender of children (only boys, p < 0.001; both boys and girls, p = 0.019). Furthermore, problem-focused coping was significantly predicted by age (p = 0.004), gender of children (male child, p = 0.002; both boy and girl: p = 0.014), and avoidant-focused coping (p < 0.001).

**Conclusions:**

The study examined how postpartum women cope with the challenges of motherhood and how this relates to their demographic and maternity factors. The results suggest that healthcare professionals should promote effective coping strategies and discourage avoidance-oriented approaches in postpartum interventions.

## Background

Motherhood is not always a simple adjustment and may be stressful, whether nurturing the first or second child [1]. Postpartum depression is a frequent mental health issue that affects women after giving delivery, and it affects many women due to this stressful phase, it is a frequent mental health issue that affects many mothers following delivery. It can have a detrimental impact on a mother’s health, the degree of the mother-infant bond, and the child’s development [2]. Therefore, it’s critical to pinpoint the risk variables associated with Coping Strategies in Postpartum Women. Women are particularly sensitive to depression in the postnatal period, with depression impacting 10-20% of mothers within the first year following birth. Nevertheless, only half of the women who exhibit significant symptoms are given a diagnosis [3].

Investigations on encountered stress show a major connection relationship between postpartum stress levels as indicated by mothers and stressful life situations experienced during pregnancy [4]. According to a recent study by Jayaseelan et al. (2020) on postpartum moms’ coping mechanisms, emotional coping mechanisms are employed more frequently than problem-focused mechanisms. The infant’s physical state and emotional coping mechanisms were significantly correlated. However, not all women adopt coping mechanisms in the same manner or to the same degree [5]. Numerous demographic characteristics, such as age, education, income, marital status, parity, and job position, may influence the choice and efficacy of coping mechanisms. In fact, research by Walker and Murry (2022) discovered that younger women in the prolonged postpartum period reported greater stress and employed more emotion-focused coping than older mothers [6]. In accordance with a study in Saudi Arabian women with lower education levels are more likely to have postpartum depression than those with higher education [7].

The mode of delivery, or how mothers felt about their childbirth experience, is a further factor that may affect the usage and success of coping methods. Some mothers may experience excitement, empowerment, happiness, and achievement after giving birth. Others could experience unpleasant emotions including dread, hurt, trauma, frustration, or a lack of control. For instance, Zanardo et al. (2020) discovered as compared to women who gave birth vaginally, those who had caesarean deliveries showed a larger inclination to utilize avoidant-focused coping techniques. Mothers’ reactions to postpartum depression and stress may be impacted by these emotions [8]. Hence, the aim of the current study is to obtain a better understanding of the coping mechanisms used by postpartum women and to investigate how various demographic and maternal characteristics affect these mechanisms.

## Methods

### Study aim

The aim of the study is to gain insight into the coping strategies employed by postpartum women and to explore how these strategies are influenced by various demographic and maternity factors.

### Study design

The study adopted a quantitative, cross-sectional design, which was conducted to achieve the aim of the study. Cross-sectional studies are appropriate for describing the phenomena at a fixed time, easy to conduct, and quick (no maturation, risk for mortality, and no long follow-up period) [9]. Data was collected from 239 women using the Brief COPE survey [10] and the socio-demographics and obstetric/maternal history form.

### Setting

This study has been conducted among mothers in an urban area of the Eastern Province in Saudi Arabia. It was conducted at the Gynecology and Obstetrics Clinic in King Fahad Hospital of the University in the eastern province of Saudi Arabia, which provides high-quality services in Obstetrics and Gynecology.

### Sampling

The study used a convenience purposive sampling technique. Participants were recruited from King Fahad Hospital of the University during their visit to the Gynecology and Obstetrics Clinic within six weeks after childbirth. The potential participant was nominated through nurses working at the outpatient clinic and given an invitation letter with complete information about the study, a web link, and a barcode for study instruments—interested participants filled in a self-reported questionnaire electronically. Data were collected between October 2022 to April 2023.

Sample size calculation was obtained based on the World Health Organization [11] at a 95% confidence level, margin of error of 0.05, and 80% response rate; therefore, the needed sample size of 235 participants was sufficient for the current study. All types of deliveries were included in the study. The eligibility criteria were as follows: women aged between 18 and 49 had childbirth and are currently postpartum.

### Ethical considerations

All study procedures were approved by the Institutional Review Board at Imam Abdulrahman Bin Faisal University (IRB-2022-04-435). The participants were assured that their contributions, information, and records would be treated as confidential and that their participation was voluntary. Participants were assured that their participation would not affect the hospital care that they received. Eligible women were given information sheets, and those who agreed to participate signed the consent before they were interviewed. The privacy of the electronic survey was ensured by using password-protected storage.

### Study tools

The researchers collected the data using the following tools:

- **The Brief COPE**: was used to measure the coping strategies among postpartum women. The Brief COPE is a shortened version of the COPE scale that was originally developed to measure coping with any stressor [10]. It is a self-reported survey comprising 28 items that assess the degree to which a respondent utilizes a specific coping strategy. The 28 items have been divided into 14 sub-scales, named active coping, planning, positive reframing, acceptance, humor, religion, using emotional support, using instrumental support, self-distraction, denial, venting, substance use, behavioral disengagement, and self-blame [10], and three higher order categories known as problem-focused, emotion-focused [12, 13], and avoidance or dysfunctional coping [12]. Which measures the individual’s coping with stressful situations. Alternatively, the brief COPE items have been categorized into “adaptive” and “maladaptive” coping. The response for each question was rated on a four-point Likert scale ranging from 1, “I have not been doing this at all,” to 4, “I have been doing this a lot.” Scores obtained on each subscale are interpreted concerning two overall coping styles: avoidance and approach. The Arabic version of the Brief COPE instrument was reported as valid and reliable [14]. Indeed, the brief COPE has been used in more than 400 research. In this study, after removing outliers using Mahalanobis distance, the Cronbach’s alpha coefficient was calculated to be 88%.
- **Sociodemographic and obstetric/maternal history form**: The researchers developed the Socio-demographics and obstetric/maternal history form to get information about the study participants, such as age, marital status, education level, employment status, type of family, and support person. In addition, questions related to obstetric/maternal history were included: the current delivery, previous reproductive history, pregnancy complications, and the presence of emotional or chronic illness during pregnancy. Furthermore, a question regarding traditional belief and practice application during the postpartum period was added based on the literature review.

### Data analysis

Data were stored and analyzed using the Statistical Package for the Social Sciences (SPSS) version 22 and Microsoft Excel. Categorical variables were assessed using frequency and percentage, while continuous variables were expressed using mean and standard deviation. The scale used in the study was evaluated through reliability analysis, internal consistency assessment, and calculation of Cronbach alpha coefficients. Independent t-tests were used to identify differences between two groups and one-way analysis of variance (ANOVA) was used to analyze differences between more than two groups. Multiple linear regression analysis was used to identify predictors of coping strategies. A p-value less than 0.05 was considered statistically significant.

## Results

Table 1 presents the characteristics of women in postpartum. The sample consists of 239 married women in postpartum. All of the women had completed a university degree and were currently employed. 33.1% of the women were in the age range of 21–30, 33.5% were between 31 and 40, and the remaining 33.5% were over 40 years old. The majority of the women (95.0%) had a family income of over 10,000 SAR, and their husbands were also largely employed (99.2%). The women had an average of 2–4 children, with 58.6% having 1–2 kids, 27.6% having 2–4 kids, 7.1% having 4–6 kids, and 6.7% having more than 6 kids. The majority of the women (72.8%) had both boys and girls, while 18.4% had only girls, and 8.8% had only boys. In terms of pregnancy and childbirth, most of them gave birth vaginally (90.8%). Only a small proportion of women had a cesarean Sect. (9.2%). The majority of women breastfed their newborns (76.2%), while 8.8% used formula, and 15.1% used both methods. Nearly all women had planned their pregnancy (92.5%), with only a few having unplanned pregnancies (7.5%). Only a small proportion of women had a disease during pregnancy (9.6%), while none had a history of depression.

**Table 1:** Demographic and health characteristics of married women in postpartum

Variable	Categories	Frequency	Percent
Marital Status	Married	239	100.0
Age	21–30	79	33.1
	31–40	80	33.5
	> 40	80	33.5
Mother Education	University	239	100.0
Employment	Employed	239	100.0
Husband Employment	Employed	237	99.2
	Unemployed	2	0.8
Family Income (SAR) *	5000–10,000	12	5.0
	> 10,000	227	95.0
Number of Kids	1–2	140	58.6
	2–4	66	27.6
	4–6	17	7.1
	> 6	16	6.7
Gender of Kids	Girls	44	18.4
	Boys	21	8.8
	Both	174	72.8
Type Of Newborn Feeding	Breast	182	76.2
	Bottle	21	8.8
	Both	36	15.1
	Total	239	100.0
Type of Delivery	CS	22	9.2
	NVD	217	90.8
Planned Pregnancy	No	18	7.5
	Yes	221	92.5
Disease During Pregnancy	Yes	23	9.6
	No	216	90.4
History of Depression	No	239	100.0

* SAR: Saudi riyal

Table 2 shows the results of a study on coping strategies used by individuals in different situations. The study found that emotional-focused coping was significantly higher for individuals aged over 40 (M = 3.49) compared to those aged 21–30 (M = 3.47, p = 0.002) and 31–40 (M = 3.45). Additionally, both’ gender of kids were associated with more emotional-focused coping (M = 3.48, p = 0.007) compared to boys (M = 3.44). Furthermore, type of delivery was significantly related to coping strategies, with those who had a cesarean section (M = 3.48, p = 0.001) using more avoidant-focused coping than those who had a normal vaginal delivery (M = 3.47). Lastly, individuals who planned their pregnancy did not significantly differ in their coping strategies compared to those who did not plan their pregnancy, nor did individuals who had a disease during pregnancy compared to those who did not.

**Table 2:** Association between coping strategies and demographic factors in women during pregnancy

		Problem Focused Coping			Emotional Focused Coping			Avoidant Focus Coping		
		Mean (SD)	T value	P value	Mean (SD)	T value	P value	Mean (SD)	T value	P value
Age	21–30	3.98 (0.04)	1.989	0.139	3.47 (0.07)	6.608	0.002 ^a^	2.09 (0.06)	0.833	0.436
	31–40	3.98 (0.05)			3.45 (0.07)			2.07(0.09)		
	> 40	3.99 (0.03)			3.49 (0.06)			2.07 (0.09)		
Husband Employment	Employed	3.99 (0.04)	1.470	0.143	3.47 (0.06)	-0.560	0.579	2.081 (0.8)	0.305	0.760
	Unemployed	3.94 (0.08)			3.50 (0.0)			2.06 (0.08)		
Family Income (SAR)	5000–10,000	3.98 (0.04)	0.032	0.747	3.45 (0.0)	-0.887	0.376	2.09 (0.06)	0.535	0.593
	> 10,000	3.98 (0.05)			3.47 (0.06)			2.08 (0.08)		
Number of Kids	1–2	3.98 (0.05)	1.266	0.287	3.46 (0.06)	1.352	0.258	2.07 (0.08)	0.824	0.482
	2–4	3.98 (0.04)			3.47 (0.06)			2.08 (0.09)		
	4–6	3.97 (0.05)			3.49 (0.05)			2.06 (0.09)		
	> 6	4.00 (0.00)			3.48 (0.04)			2.11 (0.04)		
Gender of Kids	Girls	3.97 (0.07)	1.721	0.181	3.46 (0.07)	5.103	0.007 ^b^	2.08 (0.8)	0.820	0.442
	Boys	3.99 (0.03)			3.44 (0.07)			2.06 (0.08)		
	Both	3.98 (0.04)			3.48 (0.06)			2.08 (0.08)		
Type of Newborn Feeding	Breast	3.98 (0.05)	1.999	0.138	3.47 (0.06)	1.047	0.353	2.08 (0.09)	1.665	0.191
	Bottle	4.00 (0.00)			3.48 (0.06)			2.11 (0.04)		
	Both	3.99 (0.02)			3.48 (0.06)			2.08 (0.08)		
Type of Delivery	CS	4.00 (0.00)	4.909	0.001	3.48 (0.05)	0.780	0.436	2.11 (0.03)	3.656	0.001
	NVD	3.98 (0.04)			3.47 (0.06)			2.07 (0.08)		
Planned Pregnancy	No	9.98 (0.04)	0.720	0.943	3.46 (0.06)	-0.786	0.433	2.09 (0.06)	0.845	0.399
	Yes	3.98 (0.04)			3.47 (0.6)			2.07 (0.8)		
Disease During Pregnancy	Yes	3.98 (0.04)	0.410	0.682	3.46 (0.08)	-0.896	0.379	2.09 (0.05)	0.680	0.497
	No	3.98 (0.04)			3.47 (0.06)			2.07 (0.08)		

^a^ Tukey HSD test: > 40 versus 21–30 & 31–40, p < 0.033 & p < 0.001, ^b^ Tukey HSD test: Both versus Boys

The results revealed that participants reported higher mean scores for problem-focused coping (M = 3.99, SD = 0.05) compared to emotion-focused coping (M = 3.47, SD = 0.06) and avoidant-focused coping (M = 2.08, SD = 0.09). The median score for problem-focused coping was 4.00, indicating that the majority of participants used this coping strategy frequently. In contrast, the median score for emotional-focused coping was 3.50 and for avoidant-focused coping was 2.13, indicating that participants used these strategies less often (Table 3).

**Table 3:** Means and standard deviations of problem-focused, emotion-focused, and avoidant-focused coping strategies

Coping Strategies	Mean	SD	Median	Minimum	Maximum
Problem Focused Coping	3.99	0.05	4.00	3.75	4.00
Emotional Focused Coping	3.47	0.06	3.50	3.17	3.58
Avoidant Focus Coping	2.08	0.09	2.13	1.75	2.13

Table 4 indicates that there was a moderate-to-strong positive relationship between the predictors and Problem COPE, as indicated by the correlation coefficient (R) of 0.624. Additionally, the table presents other statistical values, such as the R^2^ of 0.389, which indicates that the predictors explain approximately 39% of the variance in Problem COPE. The F-value of 37.134 indicates that the overall model is statistically significant, and the Adjusted R-squared value of 0.379 accounts for the number of predictors and sample size. The results suggest that women who use avoidant coping strategies are more likely to experience problem-focused coping difficulties postpartum, as Avoidant Focus Coping is a significant predictor of Problem COPE (β = 0.599, SE = 0.028, p < 0.001). The study also found that women over the age of 40 were more likely to report higher levels of problem-focused coping difficulties compared to women aged 21–30 (β = 0.149, SE = 0.005, p = 0.004). Regarding Gender, having a male child (β = 0.190, SE = 0.010, p = 0.002) or having both a boy and a girl (β = 0.148, SE = 0.006, p = 0.014) were associated with higher levels of problem-focused coping difficulties compared to having a female child. However, it is worth noting that these effects were relatively small compared to the effect of Avoidant Focus Coping.

**Table 4:** The predictors of problem focus coping among postpartum women

Variable	B	SE B	β	Sig.	R^2^	F	Adj R^2^
Constant	3.288	0.059		0.001	0.389	37.134	0.379
Avoidant Focus Coping	0.326	0.028	0.599	0.001			
Age (Ref: 21–30)							
Age > 40	0.015	0.005	0.149	0.004			
Gender (Ref: Female)							
Boys	0.031	0.010	0.190	0.002			
Boy & Girl	0.015	0.006	0.148	0.014			

Table 5 presents the results of the analysis examining the predictors of Avoidant Focus Coping among postpartum women. The table includes several variables, including Problem Focused Coping, Age, Gender of Kids, Emotional Focused Coping, and No Kids. The correlation coefficient (R) between the predictors and Avoidant Focus Coping was 63.5, suggesting a moderate-to-strong positive relationship between the variables. The table also presents other statistical values, including the R-squared (R^2^) value of 0.403, which indicates that the predictors explain approximately 40% of the variance in Avoidant Focus Coping. The F-value of 25.989 indicates that the overall model is statistically significant, and the Adjusted R-squared (Adj R^2^) value of 0.387 accounts for the number of predictors and sample size.

The results indicate that Problem Focused Coping is a significant predictor of Avoidant Focus Coping (β = 0.616, SE = 0.095, p < 0.001). This suggests that women who experience more difficulties with problem-focused coping strategies are more likely to use avoidant coping strategies postpartum. Emotional Focused Coping is a significant negative predictor of Avoidant Focus Coping (β = -0.111, SE = 0.069, p = 0.034). This suggests that women who use emotional coping strategies are less likely to use avoidant coping strategies postpartum.

Age is also a significant predictor of Avoidant Focus Coping, with women aged 21–30 reporting higher levels of avoidant coping strategies compared to women over the age of 40 (β = 0.16, SE = 0.010, p = 0.003). In terms of Gender of Kids, the results suggest that having only boys (β = -0.225, SE = 0.018, p < 0.001) is associated with higher levels of avoidant coping strategies compared to having only girls. Having both boys and girls (β = -0.146, SE = 0.012, p = 0.019) is also associated with higher levels of avoidant coping strategies. Finally, having more than six children (β = 0.106, SE = 0.018, p = 0.047) is also associated with higher levels of avoidant coping strategies compared to women who have 1–2 children.

**Table 5:** The predictors of avoidant focus coping among postpartum women

Variable	B	SE B	β	Sig.	R^2^	F	Adj R^2^
Constant	-1.908	0.432		0.001	0.403	25.989	0.387
Problem Focused Coping	1.132	0.095	0.616	0.001			
Emotional Focused Coping	-0.147	0.069	-0.111	0.034			
Age (Ref: > 40)							
21–30	0.029	0.010	0.16	0.003			
Gender (Ref: Female)							
Boys	-0.067	0.018	-0.225	0.001			
Boy & Girl	-0.028	0.012	-0.146	0.019			
Number of Kids (Ref: 1–2)							
> 6	0.036	0.018	0.106	0.047			

Table 6 displays the results of the analysis examining the predictors of Emotional Focus Coping. The table includes two variables, Age and Gender, and the correlation coefficient (R) between the predictors and Emotional Focus Coping was 0.284, suggesting a weak positive relationship between the variables. The table also presents other statistical values, including the R-squared (R^2^) value of 0.080, indicating that the predictors explain approximately 8% of the variance in Emotional Focus Coping. The F-value of 10.271 indicates that the overall model is statistically significant, and the Adjusted R-squared (Adj R^2^) value of 0.073 accounts for the number of predictors and sample size. The results suggest that being over 40 years old and having both a boy and girl as children are positively related to Emotional Focus Coping, with β values of 0.229 and 0.168, respectively.

**Table 6:** The predictors of emotional focus coping

Variable	B	SE B	β	Sig.	R^2^	F	Adj R^2^
Constant	3.464	0.005		0.001	0.08	10.271	0.073
Age (Ref: 31–40)							
> 40	0.031	0.009	0.229	0.001			
Gender (Ref: Female)							
Boy & Girl	0.024	0.009	0.168	0.008			

## Discussion

This study explored the coping strategies employed by postpartum women and their relationship with demographic and maternity factors. The findings indicate that women predominantly utilized problem-focused coping as their primary strategy for coping, followed by emotion-focused coping and avoidant-focused coping strategies as a form of adaptive coping. The present study suggests that postpartum women from Saudi Arabia are inclined towards employing problem-focused coping as a means of adjusting to their new maternal role. The present study is consistent with the research conducted by Andarini and Ulya (2022), which concluded that Indonesian mothers experiencing postpartum tended to rely on problem-focused coping [15]. A similar finding was reported in the study by Mathew et al. (2017), where the majority of Indian mothers at Aims, Kochi also employed problem-focused coping strategies [16].

On the contrary, many studies have found mothers to utilize emotion-focused coping strategies in order to adapt, especially when experiencing postpartum blues and/or postpartum depression [5, 17–19]. A recent content analysis conducted by Kim et al. (2022) among South Korean mothers revealed that the majority of them employed emotion-focused coping strategies [18]. Similarly, Jayaseelan and Mohan (2020) found that the majority of Indian women relied on emotion-focused coping strategies, which were associated with higher levels of stress compared to those who utilized problem-focused coping strategies [5]. A study in Ethiopia found a significant difference in coping strategies between women from urban and rural areas, with the former more likely to use problem-focused coping, while the latter tended to employ emotion-focused coping strategies [17].

Mothers might choose to cope using certain coping strategies based on a number of factors including personality, the nature of the stressors, and the availability of different resources [17, 20]. The disparities observed in the results of this study when compared to previous literature can be attributed to the unique sociocultural context of the Saudi society. Notably, the use of problem-focused coping strategies has been linked by researchers with the availability of resources that a mother can access, including support from individuals in her immediate relatives. This factor may explain the significant reliance on problem-focused coping strategies among Saudi mothers, given the family-oriented nature of Saudi society and the likelihood of new mothers being surrounded by a reliable support system [21]. In many instances, mothers in Saudi Arabia typically reside with their immediate families, including parents and siblings, which provides them with a sense of reassurance throughout the initial weeks of the postpartum period. This is unlike in Western societies, which prioritize individualism, which implies that women may experience difficulty in obtaining the necessary support during such a critical period [22]. Mothers are more likely to adopt problem-focused coping strategies when adequate resources are available to them and when they are able to make the necessary adjustments to cope with the changes they are facing. This stands in contrast to the utilization of emotion-focused and avoidant-focused coping strategies, which mothers tend to resort to when they are unable to adapt to the new changes; consequently, rely heavily on their internal emotions or avoid confronting the problem altogether [23].

Furthermore, the study demonstrated that women who relied on avoidant coping strategies were more prone to facing difficulties with problem-focused coping during the postpartum period. Relying on avoidant coping strategies indicates poor adaptability to change [24, 25]. This is in contrast to the utilization of problem-focused coping, in which mothers seek support and employ available resources to fulfill their needs. The utilization of problem-focused coping is deemed to be more effective as a coping strategy that has been associated with diminished levels of stress and enhanced mental well-being [26]. On the other hand, individuals who adopt emotion-focused coping are more vulnerable to experiencing stress and depression, as opposed to those who utilize a problem-focused coping approach [27]. Overall, the postpartum period is characterized by numerous challenges, stressors, and demands, and therefore requires appropriate coping strategies. The absence of social and personal resources has been linked to the adoption of maladaptive coping strategies [28, 29].

The coping strategies in this study significantly correlated with age, gender of the offspring, and mode of delivery. Specifically, women aged 40 years and above, as well as those with male or both gender children, reported experiencing more substantial difficulties with problem-focused coping compared to younger women and those with only female children. The findings are consistent with previous research in the field [16, 30–32]. However, there is a scarcity of research examining the relationship between maternal age and coping strategies utilized during the initial weeks of postpartum. Prior studies have suggested that younger mothers, particularly those with ill children, are more likely to rely on emotional coping as opposed to problem-focused coping strategies. For instance, one study reported a significant negative correlation between young mothers’ age and the adoption of emotion-focused coping strategies among Iranian mothers [31]. In another study among mothers from Malaysia, where the majority of the sample (43%) comprised, women aged between 36 and 40 years, the researchers found that the majority of participants relied on emotion-focused coping strategies [32]. The observed phenomenon may be age-related to declines in adaptive capacity, which is known to diminish with increasing age, potentially impacting the individual’s ability to cope with change [30]. The gender of the child was found to play a partial role in the mother’s adaptability, particularly in cases where the mother had both male and female children. This finding may be linked to the family structure, where the presence of multiple children could potentially increase the demands placed on the mother, thus providing a plausible explanation for this finding [33].

The present investigation revealed that women who underwent caesarean delivery exhibited a greater tendency for utilizing avoidant-focused coping as compared to those who had a vaginal delivery. This finding is in line with previous research, which has demonstrated a relationship between delivery mode and coping strategies employed not only by mothers but also extending to fathers [34]. This observation can be attributed to the fact that caesarean delivery is often associated with higher rates of anxiety, stress, and depression. In a national study involving 12,619 mothers in the United States, researchers reported that mothers who underwent caesarean delivery had a significant risk of experiencing stress [35]. In fact, several studies have long established that caesarean delivery, particularly in emergency cases, can be a predisposing factor to posttraumatic stress [36, 37]. This stress may account for the deployment of maladaptive coping strategies by mothers depending on the mode of delivery.

### Recommendation and implication for practice

This study has identified how women cope during the postpartum phase, taking into account their demographics and maternity variables. It has been revealed that participants mostly relied on problem-focused coping, followed by emotion-focused coping and avoidant-focused coping. Therefore, it seems crucial for stakeholders and healthcare providers to support pregnant women by implementing education programs concerned about stress and postpartum depression. Also, it is important to consider the coping mechanism strategy that can be applied depending on the demographic and maternal factors. An implication of this is the possibility of designing usable apps about postpartum depression and sending messages of supporting and coping strategies through this application.

Future research studies are required to better understand other factors rather than demographic and maternal factors that influence the coping strategies of postpartum depression. Environmental and cultural factors play a role in the coping mechanism. Involving the service users in such research will enhance the result and support its implication.

### Limitation

There are some limitations of this study. Choosing only one hospital for data collection makes it difficult to generalize the results within Saudi Arabia. A further limitation is that it used a cross-sectional design that is in one point of time measured. Additionally, the study was conducted by self-reported questions only where participants responded either with underestimation or above estimation of their feelings.

## Conclusion

The study findings indicate that postpartum women over 40 tend to use more emotional-focused coping. Additionally, both genders of children were associated with more emotional-focused coping. Cesarean section delivery was associated with more avoidant-focused coping than normal vaginal delivery. In addition, this study identified the predictors of avoidant-focus coping and problem-focused coping. Policymakers and healthcare professionals should tailor and implement programs that might enhance coping mechanisms among postpartum women.

## Data Availability

Research data is available from the corresponding author upon request.

## Abbreviations

SAR: Saudi Riyal

## References

[1] Copeland DB, Harbaugh BL (2019). It’s hard being a mama: validation of the maternal distress Concept in becoming a mother. J Perinat Educ.

[2] Almuqbil M, Kraidiye N, Alshmaimri H, Ali kaabi A, Almutiri A, Alanazi A (2022). Postpartum depression and health-related quality of life: a Saudi Arabian perspective. PeerJ.

[3] Elsanti D, Sumarmi N. The effect of stress and social support among postpartum depression women in Indonesia. GSTF J Nurs Health Care (JNHC). 2016;3(2).

[4] Qobadi M, Collier C, Zhang L (2016). The Effect of Stressful Life events on Postpartum Depression: findings from the 2009–2011 Mississippi pregnancy risk Assessment Monitoring System. Matern Child Health J.

[5] Jayaseelan J, Mohan M (2020). Coping strategies used by postnatal mothers with perceived stress. Indian J Psychiatry.

[6] Walker LO, Murry N (2022). Maternal stressors and coping strategies during the extended Postpartum Period: a retrospective analysis with contemporary implications. Women’s Health Reports.

[7] Alzahrani J, Al-Ghamdi S, Aldossari K, Al-Ajmi M, Al-Ajmi D, Alanazi F (2022). Postpartum Depression Prevalence and Associated factors: an observational study in Saudi Arabia. Med (B Aires).

[8] Zanardo V, Angelini S, Ajao ST, Cimento O, Maione R, Giliberti L (2020). First-time fathers’ coping strategies at elective cesarean delivery: a quantitative study. Early Hum Dev.

[9] Polit D, Beck C, Polit D (2017). Esource manual for nursing research, generating and assessing evidence for nursing practice.

[10] Carver CS (1997). You want to measure coping but your protocol’ too long: consider the brief cope. Int J Behav Med.

[11] WHO. https://cdn.who.int.2023. Sample size calculator.

[12] Carver CS, Scheier MF, Weintraub JK (1989). Assessing coping strategies: a theoretically based approach. J Pers Soc Psychol.

[13] Folkman S (1984). Personal control and stress and coping processes: a theoretical analysis. J Pers Soc Psychol.

[14] Hammoury N, Khawaja M, Mahfoud Z, Afifi RA, Madi H (2009). Domestic Violence against women during pregnancy: the case of Palestinian refugees attending an Antenatal Clinic in Lebanon. J Womens Health.

[15] Andarini A, Ulya Z, COPING STRATEGY IN, MOTHER WITH POST PARTUM BLUES EXPERIENCE IN KEDIRI (2022). A CASE REPORT. J Psychiatry Psychol Behav Res.

[16] Mathew RM, Anju Philip T, Sreejamol MG (2017). Perceived postpartum stress and coping strategies among postnatal mothers at aims, Kochi. Asian J Pharm Clin Res.

[17] Azale T, Fekadu A, Medhin G, Hanlon C (2018). Coping strategies of women with postpartum depression symptoms in rural Ethiopia: a cross-sectional community study. BMC Psychiatry.

[18] Kim HS, Chung MY, Rhee ES, Kim Y (2022). Is it reciprocating or self-serving? Understanding coping strategies for postpartum depression in an online community for Korean mothers. Health Care Women Int.

[19] Habibeh S, Masumeh S, Abbas A, Alireza A (2013). A comparison of Postpartum Depression among low-risk-pregnant women with emotion- and problem-focused coping strategies. Qom Univ Med Sci J.

[20] Lazarus RS, Folkman S. Stress, Appraisal, and coping. Springer publishing company; 1984.

[21] Champion D. The paradoxical kingdom: Saudi Arabia and the momentum of reform. Columbia University Press; 2003.

[22] Lansford J, Zietz S, Al-Hassan S, Bacchini D, Bornstein M, Chang L (2021). Culture and Social Change in Mothers’ and fathers’ individualism, collectivism and parenting attitudes. Soc Sci.

[23] Guardino CM, Dunkel Schetter C (2014). Coping during pregnancy: a systematic review and recommendations. Health Psychol Rev.

[24] Kim J, Jeong Yeob Han, Shaw B, McTavish F, Gustafson D (2010). The roles of Social Support and coping strategies in Predicting Breast Cancer patients’ Emotional Well-being. J Health Psychol.

[25] Skinner EA, Zimmer-Gembeck MJ (2007). The development of coping. Annu Rev Psychol.

[26] Penley JA, Tomaka J, Wiebe JS (2002). The association of coping to physical and psychological health outcomes: a meta-analytic review. J Behav Med.

[27] Chiavarino C, Rabellino D, Ardito RB, Cavallero E, Palumbo L, Bergerone S (2012). Emotional coping is a better predictor of cardiac prognosis than depression and anxiety. J Psychosom Res.

[28] Tindle R, Hemi A, Moustafa AA (2022). Social support, psychological flexibility and coping mediate the association between COVID-19 related stress exposure and psychological distress. Sci Rep.

[29] Orzechowska A, Zajączkowska M, Talarowska M, Gałecki P (2013). Depression and ways of coping with stress: a preliminary study. Med Sci Monit.

[30] Chen Y, Peng Y, Xu H, O’Brien WH (2018). Age differences in stress and coping: Problem-focused strategies mediate the Relationship between Age and positive affect. Int J Aging Hum Dev.

[31] Shavaki MA, Harandi TF, Pourabbasi A, Rahimzadeh M (2018). Coping strategies in Iranian mothers of children with type 1 Diabetes. J Diabetes Metab Disord.

[32] Salleh NHM, Mokhtar DMM (2022). Coping strategies and help seeking behavior among women with symptoms of Postpartum Depression in Selangor. Malaysian J Med Health Sci.

[33] Taylor JY, Washington OGM, Artinian NT, Lichtenberg P, PARENTAL STRESS AMONG AFRICAN, AMERICAN PARENTS AND GRANDPARENTS (2007). Issues Ment Health Nurs.

[34] Zanardo V, Manghina V, Giliberti L, Vettore M, Severino L, Straface G (2020). Psychological impact of COVID-19 quarantine measures in northeastern Italy on mothers in the immediate postpartum period. Int J Gynecol Obstet.

[35] Chen HH, Lai JCY, Hwang SJ, Huang N, Chou YJ, Chien LY (2017). Understanding the relationship between cesarean birth and stress, anxiety, and depression after Childbirth: a nationwide cohort study. Birth.

[36] Orovou E, Dagla M, Iatrakis G, Lykeridou A, Tzavara C, Antoniou E (2020). Correlation between Kind of Cesarean Section and posttraumatic stress disorder in Greek Women. Int J Environ Res Public Health.

[37] Ryding EL, Wijma B, Wijma K (1997). Posttraumatic stress reactions after emergency cesarean section. Acta Obstet Gynecol Scand.

